# Cotracking of Na^+^ and Br^–^ Adsorption through Surface-Induced Quadrupolar Relaxation (SIQR): An Alternative to Zeta Potential Measurements for High Ionic Strengths (>0.1 M) and Nondispersed Liquid–Solid Mixtures

**DOI:** 10.1021/acs.jpca.5c02542

**Published:** 2025-09-15

**Authors:** Zlanseu Ruth Tan, Cécile Pagnoux, Vincent Sarou-Kanian, Sandra Ory, Michaël Deschamps

**Affiliations:** † CEMHTI, CNRS UPR 3079, Université d’Orléans, Orléans F-45071, France; ‡ IRCER, Centre Européen de la Céramique, Université de Limoges, 12 rue Atlantis, Limoges 87068, France; & Réseau sur le Stockage Electrochimique de l’Energie (RS2E), CNRS FR3459, Cedex 1, Amiens 80039, France

## Abstract

We compare traditional zeta potential measurements with a surface-induced quadrupolar relaxation-based approach to characterize the surface electric properties of oxides in aqueous media, particularly at high ionic strengths and solid contents where classical methods fail. Three oxide materials (two TiO_2_ and one α-alumina) were tested in 1 M NaBr aqueous solutions. Zeta potential measurements are expected to yield reliable isoelectric points (IEP) at moderate solid contents and up to 0.1 M ionic strengths. In contrast, NMR relaxation rate measurements, exploiting surface-induced quadrupolar relaxation (SIQR) of ^81^Br and ^23^Na, successfully provided IEP even at 1 M ionic strength and high solid content, although they are usually much more challenging under conditions suitable for conventional zeta potential measurements. Our NMR results correlated well with zeta potential trends at lower concentrations. Notably, TiO_2__325 showed only acidic surface behavior, likely due to surface carbonate and/or phosphate groups. Surface acidity constants derived from NMR data allowed IEP estimation consistent with the literature values. This demonstrates that NMR relaxation rate measurements provide a robust alternative to zeta potential experiments under conditions where the latter are unreliable

## Graphical abstract

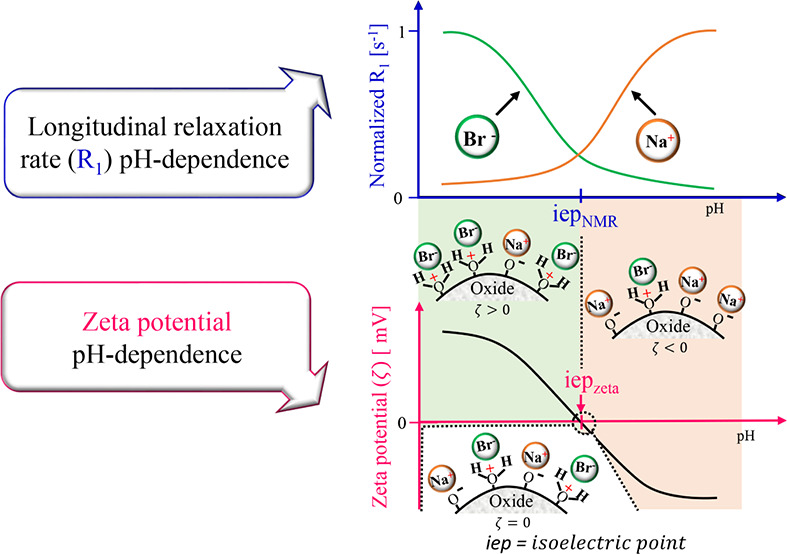

## Introduction

To characterize the surface electric properties of metallic oxides in aqueous media, researchers often refer to electrokinetic techniques.^1–5^ This generally results in the determination of the zeta potential (ζ), which is defined as the average potential at the shear plane, according to the electric double layer theory.^6^ The zeta potential is of great interest for many scientific fields, since it is, for instance, a good predictor of the surface net charge (σ_0_) of materials in solution^6–8^ and generally governs the affinity between charged or polar species and particles in solid/liquid mixtures.^7,9–11^ For ionic ligands, positive or negative ζ involves the predominant adsorption of, respectively, negative and positive counterions in the Stern layer.^12^ The sign and magnitude of the zeta potential (and thus σ_0_) are influenced by a number of parameters, the main one being pH.^11^ In the specific case of metallic oxides, the effects of pH on σ_0_ are associated with the protonation or deprotonation of surface hydroxyl groups (≡M–OH) and/or the physical trapping of ions at the surface.^4,13–18^ The ionization of ≡M–OH can be illustrated by the following acid–base equilibria:^18,19^

1 $$≡M-OH+H^{+}\to ≡M-OH_{2}^{+}\;Ka_{1}$$

2 $$≡M-O^{-}+H^{+}\to ≡M-OH\;Ka_{2}$$

where ≡M–OH_2_^+^ and ≡M–O^*–*^ are the positive and negative surface sites, respectively. *K*a_i_ (i = 1 or 2) represents the acidity constant (p*K*a_i_ = −log*K*a_i_) of the *i* ^th^ surface acid–base reaction. For known p*K*a_i_, the isoelectric point can be estimated according to Equation 3:^20^

3 $$IEP=\frac{1}{2}\left(pKa_{1}+pKa_{2}\right)$$

This quantity refers to the configuration where there are as much positive as negative ionized groups (such as ≡M–OH_2_^+^ and ≡M–O^*–*^). The IEP induces the cancellation of ζ, when the latter is measured as a function of pH.^16^ In real polycrystalline samples without facet controls, surface oxygens are likely inequivalent, leading to an ensemble of p*K*a_i_ values.^21,22^

Despite the popularity of ζ-based techniques, many limitations remain. The determination of ζ is, first, subject to several assumptions as a value theoretically deduced from experimental quantities such as electrophoretic mobility, streaming or sedimentation potentials, electro-osmosis flow, or electrokinetic sonic amplitude (ESA).^23–25^ It also provides a global overview of the surface net charge, without distinguishing the intrinsic contributions of positive and negative surface charges. Moreover, zeta potential measurements remain practically challenging for samples with both high ionic strength (>0.01 M)^2,26^ and high solid content.^27^ These two latter limitations motivated the present investigations.

Our objective is to determine whether SIQR effects yield results consistent with zeta potential measurements under conditions of high ionic strength and solid content, where ESA measurements become less reliable, and to demonstrate the value of adopting a relaxometry-based approach. On the one hand, the zeta potential was measured on colloidal suspensions made of 3 wt % solid content and 0.1 and 1 M ionic strengths. The larger ionic strengths (i.e., >0.01 M) require the removal of the contribution of the background electrolyte.^9,11,28^ On the other hand, the NMR samples are compact slurries at salt concentrations ranging between 0.1 and 1 M.

NMR probes local structures at atomic resolution, as well as dynamics at different length scales,^29–33^ which makes it a suitable tool to differentiate ions according to their location and dynamics in solid/liquid mixtures. In NMR, the longitudinal relaxation rate (*R*_1_ = 1/*T*_1_) characterizes the return to equilibrium of the magnetization and is known to be sensitive to solid/liquid interface phenomena (i.e., the SIQR effect), as shown in a large number of studies in which the effects of surface charges, ionic strengths, or solid contents on ion binding properties were examined.^31,34–41^ NMR relaxation is governed by stochastic fluctuations of spin interactions due to atomic motions. The latter is related to correlation times (*τ*_c_) characterizing these motions in the medium.^29,42–44^ For the ^23^Na and ^81^Br nuclei (*S* = 3/2), relaxation is dominated by the interaction between the nuclear electric quadrupolar moment and the electric field gradient (EFG) at the nucleus.^34,35,43,45,46^ The quadrupolar relaxation is most efficient when the correlation function of the EFG fluctuations is characterized by a correlation time τ_c_ close to the inverse of the Larmor frequency $\frac{1}{\omega_{0}}$ . At a Larmor frequency close to 100 MHz, for example, dynamics of the order of nanoseconds will contribute the most to longitudinal relaxation.^47^ In water, the longitudinal relaxation of quadrupolar spin-bearing ions stems from the fast, subnanosecond motion of water molecules in the first solvation shell, which affects the electronic cloud symmetry and creates fluctuating EFG at the nucleus. In oxide/aqueous solution mixtures, the magnitude of the EFG is generally greater at solid/liquid interfaces due to the surface electric charges and the loss of symmetry in the environment.^36,48–50^ Moreover, the fluctuation of this EFG at the appropriate frequency $\left(∼\frac{1}{\tau_{c}}\right)$ is ensured by the slowdown of ion dynamics in the vicinity of oxide particles.^16^ The consequence is a relaxation that becomes much faster for the nuclear spins of counterions compared to those in the bulk, i.e., the SIQR effect.^34^ Moreover, the SIQR effect is expected to broaden the line widths of quadrupolar nuclei (in our study ^23^Na, ^81^Br, and ^35^Cl in Maki’s study).^39,51^

Comparing the SIQR effects on cations and anions is expected to provide new insights into the relative proportions of positive (≡M–OH_2_^+^) and negative (≡M*–*O^–^) surface charges. This strategy was explored on three different oxides, namely, two titanium dioxides (TiO_2__P25 and TiO_2__325) and one alpha-alumina sample (AKP-30). The electrolytes were sodium bromide (NaBr) aqueous solutions used to study ^23^Na and ^81^Br NMR relaxation as a function of pH.

## Materials and Methods

### Materials and Reagents

To implement our comparative study between zeta potential measurements and NMR SIQR effects, three model materials were chosen, namely, two titanium dioxide (TiO_2_, Sigma-Aldrich) and one alpha alumina (α-Al_2_O_3_, Sumitomo Chemical Company, Japan). Each of them was used as provided, without further purification. The first TiO_2_ sample (Degussa P25), very popular in the literature for its high photocatalytic activity,^52–55^ is a mixture of anatase and rutile polymorphs, to the extent of 70–85% and 15–30%, respectively. The second TiO_2_ is a commercial-grade, pure 325-mesh anatase powder (99.6%), which is very little described in the literature to the best of our knowledge. Thereafter, the former will be designated TiO_2–_P25 and the later TiO_2__325. Regarding the last material, it is a high-purity (>99.99%) α-Al_2_O_3_ referenced as AKP-30 (or AKP30) by the supplier and extensively used in the field of colloids and ceramics.^2–4,56,57^ Table 1 summarizes the main features of each material. While the densities and densities of surface hydroxyl groups are very similar, one can notice significant changes in the specific areas and the grain sizes. TiO_2__P25, which has the smallest grain sizes, has a specific area about four and eight times larger than TiO_2__325 and AKP-30, respectively. The grain sizes were estimated from scanning electron microscopy (SEM) images prior to the study (Supporting Information, Section A). Since we did not perform laser granulometry experiments to assess the average grain size, we provide instead size ranges obtained from SEM images. The specific areas were determined by Brunauer–Emmett–Teller (BET) analyses and found to be consistent with the literature for TiO_2__P25 ^58^ and AKP-30.^3^ Lastly, the densities of surface hydroxyl groups were derived from thermogravimetric (TGA) analyses (Supporting Information, Section B).

**Table 1:** Densities, Specific Area, Grain Sizes, and Surface Hydroxyl Group Densities for TiO_2–_P25, TiO_2__325, and AKP-30^a^

Samples	Density (g/cm^3^)	Specific area (m^2^/g)	Grain size range (nm)	Surface −OH density (σ_OH_) (/nm^2^)
TiO_2__P25	4.26	50–57	25–51	4.9
TiO_2__325	3.90	14	70–240	-
AKP-30	3.98	7	87–814	5.3

a The densities are those provided by the suppliers. The specific areas were determined by Brunauer–Emmett–Teller analyses, and the range of grain sizes was determined from the analysis of scanning electron microscopy images. The last column represents the density of surface hydroxyl groups derived from thermogravimetric analyses.

### Sample Preparation for NMR Experiments

For each oxide, two sets of samples were prepared for the NMR SIQR experiments. The first set contained samples with variable ionic strengths at a constant pH value, and the second set consisted of samples with variable pH at a constant ionic strength.

To prepare the variable ionic strength samples, appropriate amounts of NaBr (Sigma-Aldrich) were first dissolved in ultrapure water (Milli-Q) to obtain solutions with concentrations ranging from 0.1 to 1 M. For the variable pH samples, an aqueous NaBr solution at the optimum concentration was prepared using the optimized ionic strength obtained from the preliminary study. From this primary solution, we derived a set of acid–base solutions by adding gradual amounts of HBr or NaOH (1 M, Sigma-Aldrich) to obtain solutions with pH_0_ values ranging from 2.3 to 12.4. We neglected the influence of the added volumes used to adjust the pH on the ionic strength, assuming that the ionic strengths remained unchanged after pH adjustment.

For the remaining steps, the procedure is the same for both the variable ionic strengths and the variable pH of oxide/NaBr mixtures. The NaBr solutions (either variable ionic strengths or variable pH) were mixed with each oxide up to a 3 wt % solid content. The mixtures were kept under stirring at 500 rpm for a minimum of one h to allow equilibration. The initial pH_0_ and the final pH of the mixtures after equilibration (pH_mi*x*_) were measured using a SevenCompact S210 Mettler Toledo pH meter. Prior to the measurement, the pH meter was calibrated with commercial-grade solutions of pH 4.01, 7.00, and 9.21. After equilibration, each mixture was filtered with a nylon membrane filter of 0.45 μm diameter (Merck, France), and the supernatants were separated from the sediments. The latter were weighed and transferred into a 10 mm NMR quartz tube. The quartz tubes containing the sediments were then centrifuged at 4000 rpm for 6 min (G-force of 2254 RCF) with an EBA 270 centrifuge (Hettich, Germany) to properly pack the samples. It was assumed that the pH values in the sediments were the same as those measured in the prefiltration mixtures (i.e., pH_mix_). Through a straightforward calculation procedure detailed in Supporting Information, Section C, we estimated the solid contents to reach 41, 72, and 86 wt % in the sediments involving TiO_2__P25, TiO_2__325, and AKP-30, respectively.

### Zeta Potential Measurements

Zeta potentials as a function of pH were measured for TiO_2__P25, TiO_2__325, and AKP-30 using an ESA analyzer (AcoustoSizer II S flow-through system, Colloidal Dynamics). The oxide powders were first dispersed into reverse osmosis water at 3 wt % solid content, and appropriate amounts of NaBr were added to achieve ionic strengths of either 0.1 or 1 M. The mixtures were ultrasonicated (Vibra-Cell ultrasonic disintegrator VC 600, Sonic & Materials, USA) to deagglomerate the powders. The corresponding NaBr aqueous solution was used as the background electrolyte. Two suspensions were prepared for each oxide to cover the pH range from 3 to 11. A pH sensor, included in the measuring equipment, automatically monitored acid–base titrations. The acidic and basic pH levels were adjusted with 1 M aqueous solutions of HBr and NaOH during titration. The natural pH (without the addition of any acid or base) of TiO_2__P25, TiO_2__325, and AKP-30 in 0.1 M NaBr aqueous solutions was 5.1, 6.3, and 7.7, respectively.

The electroacoustic method used by the AcoustoSizer apparatus to estimate the zeta potential has already been described in previous works.^3,4,7,59^ Briefly, a high-frequency alternating electric field (1 MHz) is applied to the colloidal suspension, which causes particle displacement due to their nonzero net charge and generates an acoustic wave at the same frequency. As the oscillating motion of particles takes place at the shear plane of the electric double layer, the resulting ESA signal is directly related to the zeta potential. The ESA is derived from an electric signal measured by a piezoelectric transducer integrated into AcoustoSizer. This quantity is proportional to the mobility of particles, which is itself related to the zeta potential through O’Brien’s formula.^24^ The final relationship between the ESA signal and the zeta potential is written as

4 $$\zeta =\frac{ESA\times \eta }{\varepsilon \times \phi \times \Delta \rho \times C}\times G\left(\alpha \right)$$

where η is the medium viscosity, ε is the solvent dielectric constant, ϕ is the solid volumetric fraction, and Δρ is the difference in density between the solid and liquid. According to O’Brien,^24^ *G*(α) is a complex corrective term that depends on the angular frequency, particle radius and density, viscosity, and dielectric constant of the solvent.

The total ESA signal is a vector sum of the signals from the suspended particles and the electrolyte ions.^26^ In the literature, zeta potentials are usually measured at ionic strengths lower than or equal to 0.01 M and low solid content (<volume fraction around 2%).^3,4^ In such a configuration, the background electrolyte signal and the particle–particle interactions are negligible, so the ESA signal is considered to reflect only the electrical properties of the isolated suspended particles.^26,60^ A background correction to the total signal has been derived to remove the ESA signal from the electrolyte (ionic strength >0.01 M),^2,26^ and another correction can compensate for the overlapping of the electric double layers in high solid content systems.^27^ The background correction option is implemented in AcoustoSizer and was used for our zeta potential measurements at 0.1 and 1 M. The following zeta potential measurements were performed on 3 wt % solid content samples to limit the effects of electric double layer overlapping on the global ESA response. Concerning the contribution of the electrolyte, the signal used for the background correction must be measured on the electrolyte alone. Ionic strengths of 0.1 and 1 M were chosen, instead of 0.01 M, to highlight the challenges related to zeta potential measurements in high ionic strength systems.

### NMR Relaxation

NMR relaxation experiments were conducted using a Bruker Avance III 400 MHz (9.4 T) spectrometer equipped with a 10 mm ^1^H/X double-resonance probe for liquid samples. At this magnetic field, the corresponding Larmor frequencies for ^23^Na and ^81^Br were 105.9 and 108.4 MHz, respectively. Since the two nuclei have close Larmor frequencies, they can typically be observed with the same NMR probehead (usually tunable to the commonly observed ^13^C nucleus), and their longitudinal relaxation exhibits similar dependencies on the correlation times of the fluctuating interactions. The longitudinal relaxation rates of ^23^Na and ^81^Br in the oxide/NaBr mixtures (sediments) were determined using the standard inversion–recovery pulse sequence. Although quadrupolar relaxation is usually biexponential^30,31,43,44,47^ in this case, only one relaxation rate was observed. The experiments consisted of 32 spectra with variable relaxation delays (*t*) ranging from 1 μs to 500 ms for ^23^Na and 1 μs to 20 ms for ^81^Br. The recycle delays were set to at least 5 × *T*_1_ for all experiments to ensure complete recovery of the magnetization between experiments. The optimal length of the π/2 pulses was 40 μs in both mixtures and pure NaBr electrolytes, regardless of the oxide, the pH, and the nucleus, confirming that these pulses excite all transitions and are not selective for the central transition. The magnetization curves were fitted using the following monoexponential function:

5 $$\frac{M_{z}}{M_{0}}=1-2I_{eff\;}\exp\left(-t/T_{1,mix}\right)$$

where *R*_1,mix_ = 1/*T*_1,mix_ is the relaxation rate of the spin in the mixtures. *R*_1,*mix*_ is optimized during the fitting procedure. *I*_eff_ is the factor that indicates the efficiency of the magnetization inversion by the π pulse in the inversion–recovery sequence. The factor *I*_eff_ is of great importance in the measurement of quadrupolar nuclei relaxation rates.^39^ If I_eff_ is lower than one (*I*_eff_ < 1), part of the magnetization undergoes relaxation during the RF pulse. The smaller *I*_eff_, the larger the portion of the magnetization that relaxes under RF irradiation. The fit was performed using the Python *curve_fit* function, and errors on the fitted *T*_*1*_ values were systematically lower than 1%. For fast-relaxing spins such as ^81^Br, long pulse durations such as those used in this work (40 μs) can be challenging, as *T*_1_ values may approach the same order of magnitude. A single *T*_1_ measurement was performed for each sample, and while performing repeated measurements would be beneficial for estimating the true experimental error.

## Results and Discussion

Figure 1 compares the noncorrected zeta potential curves as a function of pH for colloidal suspensions with TiO_2__P25, TiO_2__325, and AKP-30 at 0.1 and 1 M. For the same oxide, the curve trends are drastically modified when the concentration increases from 0.1 to 1 M. For TiO_2__P25 and AKP-30, for example, the curves that showed IEP at pH = 6.54 and 8.41 at 0.1M, no longer display them at 1 M, as the entire curves remain completely in the negative potential region over the entire pH range. In the case of TiO_2__325, the general trend remains the same, with more negative zeta values at 1 M.

**Figure 1:**
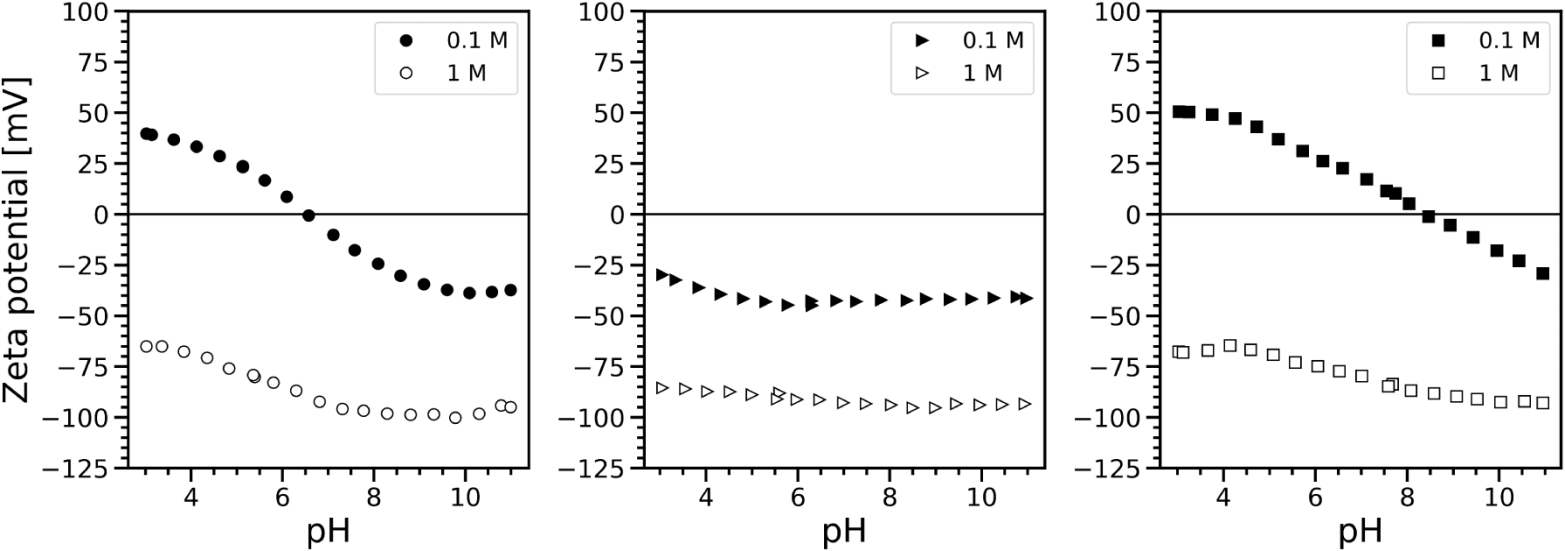
Evolution of the noncorrected zeta potential curves as a function of pH for colloidal suspensions of TiO_2__P25 (circles), TiO_2__325 (triangles), and AKP-30 (squares) at 0.1 M ionic strength. The open symbols refer to samples with the same materials at 1 M. The solid content was 3 wt % for each oxide.

This result highlights the challenge related to zeta potential measurement at high ionic strength and the necessity of background corrections. The latter was attempted for TiO_2__P25, and the result is reported below (Figure 2). After the background correction, we obtained IEP values of 7.05 and 4.53 for samples at 0.1 and 1 M, respectively. The gap between IEP at 0.1 and 1 M is however quite large, which reveals the strong influence of the ionic strength on the ESA signal. Background correction tests were also carried out on the other two materials. However, concerns have been reported regarding the reliability of measurements at high ionic strengths.^61^ Therefore, we limit our interpretation to the corrected zeta potential curves of oxides in 0.1 M ionic strength media.

**Figure 2:**
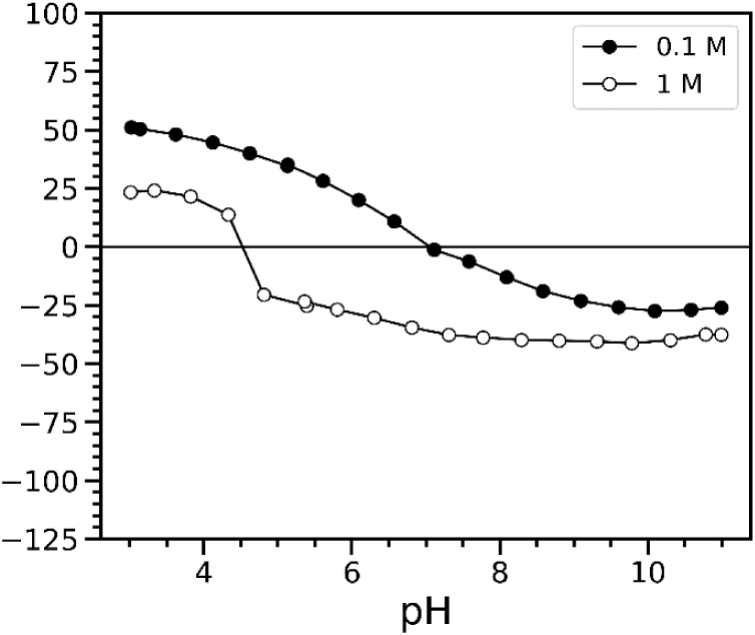
Evolution of the corrected zeta potential curves as a function of pH in a colloidal suspension of TiO_2__P25 (full symbols: 0.1 M and open symbols: 1 M ionic strengths).

Figure 3 shows the evolution of the corrected zeta potential as a function of pH for samples with TiO_2__P25, TiO_2__325, and AKP-30. This corresponds to colloidal suspensions with an ionic strength of 0.1 M and a solid content of 3 wt %. The uncertainty in measurements is about 3%. Over the entire pH range, each oxide exhibits a unique signature in terms of electric properties. The profiles of TiO_2__P25 and AKP-30 indicate IEP at pH 7.05 and 8.80, respectively, while the TiO_2__325 sample shows consistently negative zeta potentials across the entire pH range (no apparent IEP). For TiO_2__P25 and AKP-30, the positive surface net charge density (σ_0_) below IEP implies a predominant adsorption of Br^–^ ions at solid/liquid interfaces, whereas the adsorption of Na^+^ prevails above (σ_0_ < 0). The decrease of ζ with basification suggests a transition from the equilibrium between≡M–OH_2_^+^ /≡M–OH to the equilibrium between ≡M–OH/≡M–O^–^ which becomes predominant beyond the IEP. This assumption is in good agreement with the TGA and IR analyses showing the presence of surface ≡M–OH groups and the absence of surface impurities (see Supporting Information Sections C and D). The IEP at 7.05 for TiO_2__P25 is quite close to the values reported in the literature,^52,58–63^ although those have been measured at 0.01 M ionic strength. The lowest zeta potential of −27.5 mV at pH = 10.1 and the highest of 50.4 mV at pH = 3 reveal an almost amphoteric behavior for TiO_2__P25.^64,65^ AKP-30 rather exhibits a basic behavior,^16^ namely that only a weak portion of surface positive sites at acidic pH is converted into surface negative sites beyond its IEP. This IEP at pH = 8.8 is consistent with the literature, again for measurements conducted with the sample at 0.01 M ionic strength.^4^ Regarding TiO_2__325, the negative trend of the zeta potential curve is characteristic of purely acidic oxides such as SiO_2_, whose IEPs are shifted to pH values lower than 4.^16,66,67^ In the literature, such trends are very common for anatase-type^68,69^ or chemically modified TiO_2_.^63,70^

**Figure 3:**
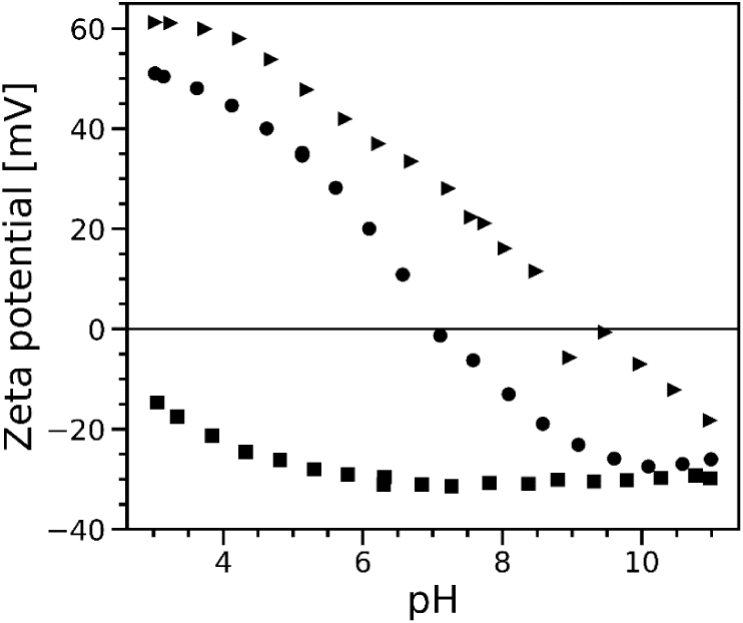
Evolution of the corrected zeta potential as a function of pH in samples made of TiO_2__325 (squares), TiO_2__P25 (circles), and AKP-30 (triangles). Oxides were dispersed in a 0.1 M aqueous solution of NaBr, with a solid content of 3 wt %. The measurement was performed by acoustophoresis, with acid/base pH adjusted with 1 M aqueous HBr and NaOH, respectively.

This behavior can be explained by the presence of acidic groups on the TiO_2__325 surface, i.e., surface groups that do not undergo protonation even at the lowest pH used here.^16,71,72^ The absence of mass loss in the temperature region associated with surface hydroxyl desorption (as seen in the TGA analysis, Supporting Information, Section B) seems to confirm the absence of M–OH groups observed in the other samples. Infrared spectroscopy, ^1^H and ^31^P NMR confirmed the presence of phosphate and/or organic groups (C=O, P=O, and P–O) on the TiO_2__325 surface (Supporting Information, Section D). The presence of phosphate groups could justify the purely acidic behavior of TiO_2__325 due to their intrinsic acidic features. Moreover, concerning the contribution of C=O groups, Kosmulski et al. have shown that the presence of carbonate (−CO_3_^2–^) induces a shift in the IEP of metal oxides to low pH.^73^ Finally, acidic oxides adsorb alkali ions according to the sequence Cs^+^ > Rb^+^ > K^+^ > Na^+^ > Li^+^, due to their ability to disrupt the structure of water in their vicinity.^5^ Such behavior has been highlighted for TiO_2__325 in our previous work,^39^ which strengthens the current assumptions.

In terms of magnitude, the oxides’ zeta potential follows the sequence AKP-30 > TiO_2__P25 in the range of positive surface charges and TiO_2__325 > TiO_2__P25 > AKP-30 in the negative charge regions. Therefore, one expects the strength of interactions between the charged particles and the counterions (Br^–^ below and Na^+^ above the IEP) to follow the same hierarchy at a given pH.

To interpret the NMR relaxation results in mixtures, fast exchange was considered between the fraction α of the spin population, which is adsorbed at the surface of oxide particles during the measurement, and the remainder in the bulk. This assumption is based on the simulation of magnetization curves, which are reproduced with a single exponential component (Figures S6 and S7). Slow exchange is expected to give rise to two distinct exponential components in the inversion–recovery curve. In the two-site fast exchange model, the observed relaxation rate $\left(R_{1,2,mix}=\frac{1}{T_{1,2,mix}}\right)$ is defined as the average of the intrinsic relaxation rates in each considered site ( $R_{1,2,surf}=\frac{1}{T_{1,2,surf}}$ and $R_{1,2,bulk}=\frac{1}{T_{1,2,bulk}}$ ), weighted by their respective populations (i.e., α and 1 – α).

This results in the following expression:^33,34^

6 $$R_{1,2,mix}=R_{1,2,bulk}+\alpha \left(R_{1,2,surf}-R_{1,2,bulk}\right)$$

In this equation, *R*_1,2,surf_ and α are the unknown quantities. *R*_1,2,bulk_ represents the relaxation of ^23^Na and ^81^Br in aqueous NaBr solutions at 1 M and variable pH. Indexes 1 and 2 refer to longitudinal and transverse relaxation, respectively. According to Equation 6, *R*_1,2,mix_ depends on both α and the relaxation rate of adsorbed species *R*_1,surf_ which are difficult to separate. Variations of *R*_1,2,mix_ can therefore be related to either variation of α,*R*_1,2,surf_ or both.

Figure 4a represents the evolution of ^23^Na longitudinal relaxation rates as a function of ionic strength, at pH ranging from [9.1 to 9.7] for TiO_2__P25, [5.8 to 6.4] for TiO_2__325, and [10.1 to 10.41] for AKP-30. The slight pH variations over the concentration ranges were disregarded in the interpretation. In the pure electrolyte systems (dashed line), the relaxation rates remain weak (*R*_1,bulk_Na_ ∼19 s^–1^) and almost unchanged over the entire concentration range.

**Figure 4:**
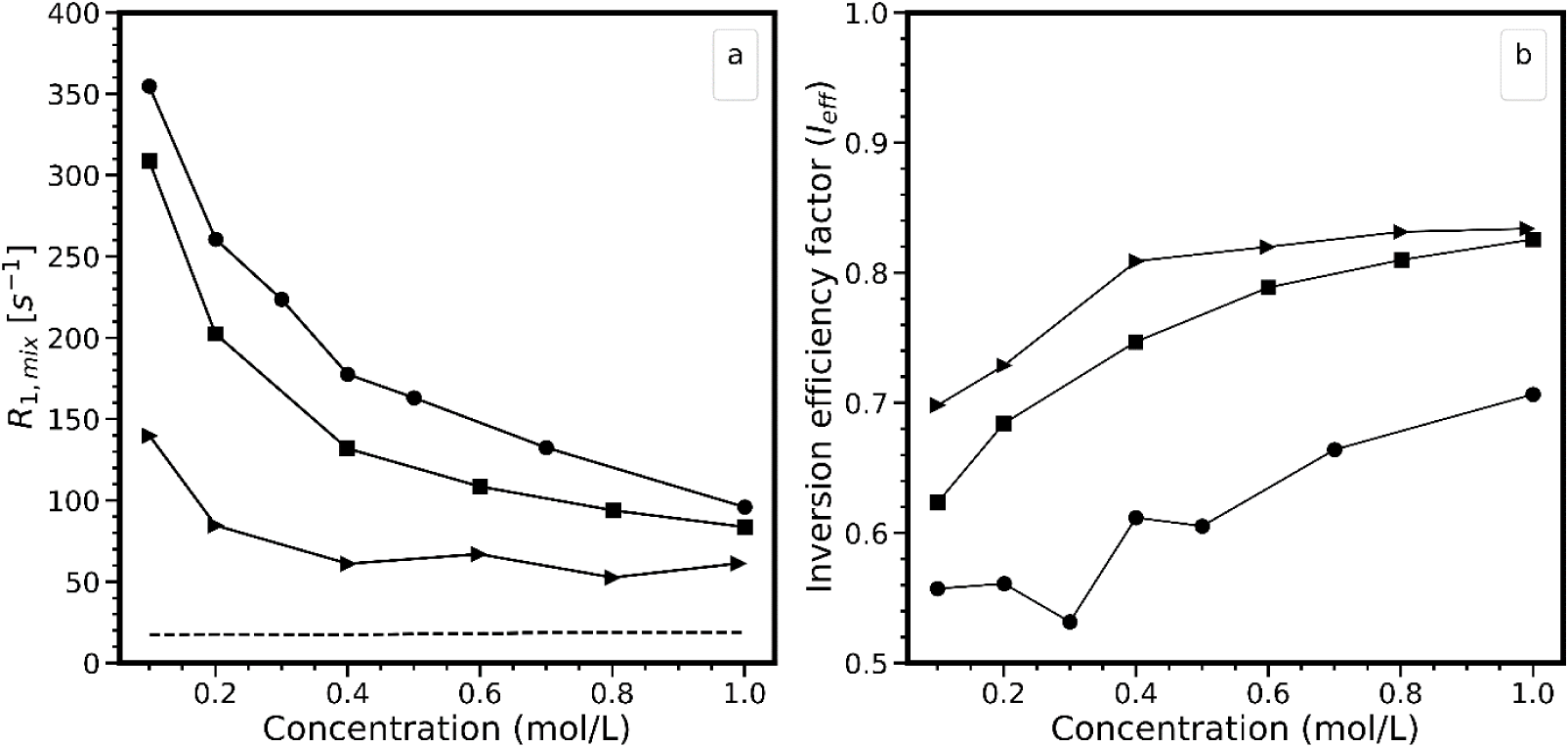
^23^Na relaxation (a) and inversion efficiency factor (b) as a function of ionic strength in systems involving TiO_2__325 (squares), TiO_2__P25 (circles), and AKP-30 (triangles). The pH ranges were 9.1–9.7 for TiO_2__P25, 5.8–6.4 for TiO_2__325, and 10.1–10.41 for AKP-30. The full lines are guides to the eyes, while the dotted line refers to the ^23^Na relaxation rates recorded in pure NaBr solutions at the corresponding ionic strengths.

This is a typical behavior of quadrupolar nuclei relaxing in aqueous solutions.^38^ This relaxation is induced by the fluctuations of the EFG, resulting from motions occurring in the water solvation shell, which is not affected by moderate changes in the electrolyte concentration.^51,74^

Regarding the oxide/NaBr mixtures, the relaxation rates increase at lower concentrations, which is also an expected result based on the works of Gossuin et al.^38^ and Kirkpatrick and coworkers.^34^ Considering the fast exchange assumption, one could explain this behavior by the decrease of the population of spins from the bulk electrolyte in favor of those adsorbed on the surface of oxide particles. As *R*_1,mix_ is a weighted average value of *R*_1,liq_ and *R*_1,surf_ (Equation 6), the weight of the latter becomes increasingly large, so that it dominates the overall relaxation phenomena. Based on these results, one could consider the concentration range below 0.5 M as the optimal one to study surface relaxation effects, as, in this concentration range, the SIQR effect is going to be stronger. However, as shown in Figure 4b, reducing the ionic strength in mixtures results in a decrease in the inversion efficiency factor. This also leads to reduced signal-to-noise ratios and longer experimental times (not shown). The maximal *I*_eff_ values are 0.7, 0.82, and 0.83 at 1 M, and the lowest are 0.5, 0.62, and 0.7 at 0.1 M for TiO_2__P25, TiO_2__325, and AKP-30, respectively. Although these values are lower than one, they remain large enough to measure *R*_1,mix_ in a precise manner (Supporting Information, Section E). A similar approach for ^81^Br in the region of positive surface charge (pH 2.7) is limited by fast signal relaxation during the RF pulses. If we consider ^81^Br relaxation in TiO_2__P25/NaBr mixtures (Figure 5), one can see that the *R*_1,mix_ results are scattered under 0.5 M, and the highest value is greater than 12000 s^–1^ at 0.1 M (i.e., *T*_1,mix_ ≈ 80 μs). In such conditions, *T*_1,mix_ is only twice as long as the π/2 pulse length (40 μs). Moreover, among the samples in this series, only the one at 1 M ionic strength exhibits an inversion efficiency (*I*_eff_) greater than 0.5. At lower ionic strengths, *I*_eff_ becomes smaller, with the lowest value around 0.3 for the 0.1 M sample. Thus, it becomes obvious that working at a low ionic strength is not the best strategy for the NMR relaxometry method developed in the current work. Moreover, lower NaBr concentrations will also lead to lower signal-to-noise ratios and longer experimental times. We therefore chose 1 M as the optimal ionic strength and preferred *R*_1,mix_ measurements to *R*_2,mix_ as the 180° refocusing pulses are unlikely to work in our systems. Our oxides are not mutually compared at this step, as our samples are not in the same conditions of pH and solid content.

**Figure 5:**
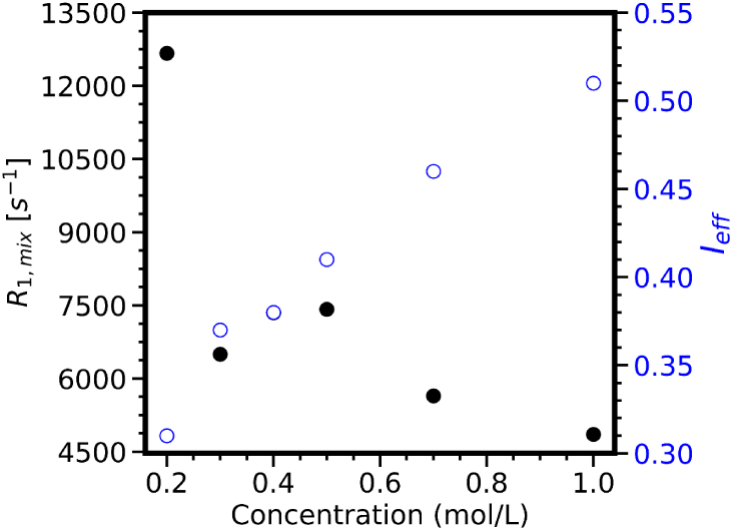
^81^Br relaxation (black full circles) and inversion efficiency factor (blue open circles) as a function of ionic strength in samples with TiO_2__P25 at a pH range of 2.71–2.92.

Based on the above discussion, the optimal ionic strength used for NMR relaxometry at variable pH is set to 1 M.

Figure 6 presents all NMR spectra recorded at each pH. All spectra exhibit a single peak at around zero ppm, with no apparent distinction between signals from adsorbed spins and those in the bulk electrolyte. Such behavior supports the fast exchange hypothesis between these two spin populations.^34^ For TiO_2__P25 and AKP-30, the spectra of ^81^Br broaden as the medium acidifies, while those of ^23^Na evolve in the opposite direction. Regarding TiO_2__325, the trend for ^23^Na is similar to what was observed for TiO_2__P25 and AKP-30, whereas no significant change over the whole pH range is observed in the corresponding ^81^Br NMR spectra. Similar effects have already been observed by Maki and coworkers for ^23^Na and ^35^Cl in mixtures made of α-Al_2_O_3_ (ζ < 0 at pH ≈<5.1 and ζ < 0 above) and SiO_2_ (ζ < 0 at pH = 2–11) dispersed in aqueous solutions of sodium chloride (NaCl, 0.1M).^36^ According to the authors, these behaviors are governed by the electrostatic interactions between oxide particles (α-Al_2_O_3_ and SiO_2_) and counterions (Cl^–^ or Na^+^) in aqueous media. In the region of positive surface charge (pH ≈<5.1 for α-Al_2_O_3_ and pH ≈<2 for SiO_2_), the particles predominantly attract Cl^–^, and the NMR lines of quadrupolar ^35^Cl ions are broadened by the SIQR effect. In the case of ^35^Cl, they observed negligible changes in the line widths for SiO_2_ slurries, as observed in our TiO_2__325. On the other hand, negative surface charges lead to the adsorption of Na^+^ ions and a broadening of their peaks, as seen in all three samples as the pH increases.

**Figure 6:**
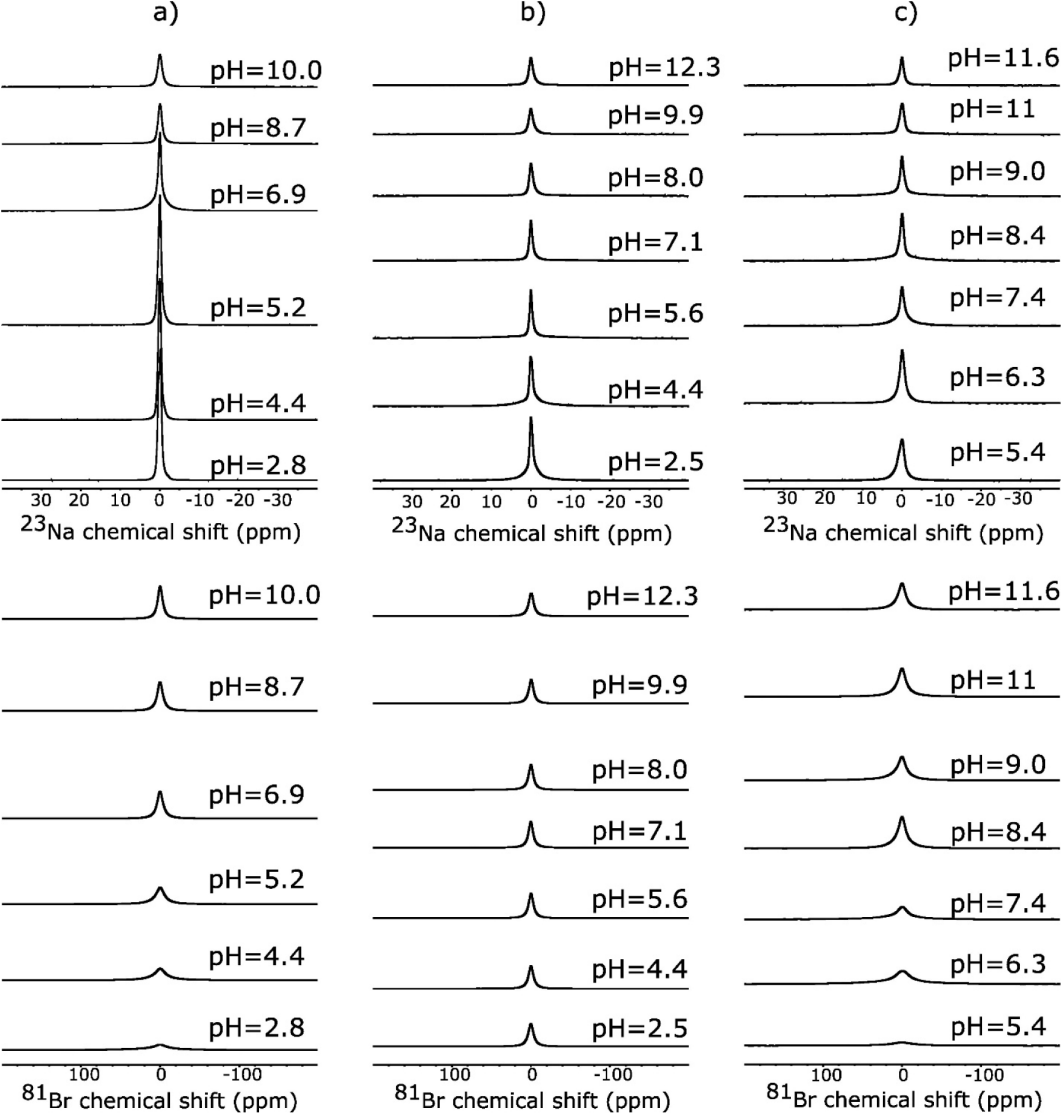
^23^Na (top) and ^81^Br (bottom) signal evolution as a function of pH in solid/liquid mixtures involving (a) TiO_2__P25, (b) TiO_2__325, and (c) AKP-30. The liquid phase consisted of a 1 M aqueous NaBr solution.

The broadening of the NMR line $\left(\propto 1/T_{2,mix}^{*}\right)$ is the earliest evidence of the SIQR effect, as the contribution of the quadrupolar relaxation to the line width of the NMR signal is proportional to the transverse quadrupolar relaxation rate.^75^ The line widths as a function of pH are presented in Supporting Information for the two TiO_2_ samples (Figure S9). However, as field inhomogeneity also affects the line widths and may depend on many uncontrollable factors, and refocusing of fast relaxation magnetization is difficult, longitudinal relaxation rate measurements were preferred in the end. As ^81^Br possesses a stronger quadrupolar coupling constant (*eQ* = 13.35 fm^2^) compared to ^23^Na (*eQ* = 10. 40 fm^2^), we systematically observed broader lines. In the same chemical environment, ^81^Br is more likely to undergo stronger SIQR effects, shorter relaxation times, and much broader spectra.

Figure 7 shows the evolution of ^23^Na and ^81^Br relaxation rates as a function of pH. The dashed line represents the longitudinal relaxation rates in pure aqueous solutions (at variable pH), while the solid lines correspond to those in mixtures composed of TiO_2__P25, TiO_2__325, and AKP-30. For mixtures involving TiO_2__P25 and AKP-30, the SIQR effect concerns ^23^Na for pH values greater than 6 and 8, respectively, while ^81^Br displays an increasing SIQR at pH values below these thresholds. Compared to the zeta potentials (Figure 3), these regions correspond to particles that are negatively and positively charged, respectively. This supports the hypothesis of electrostatic interactions between particles and counterions, as mentioned in Maki’s works^36^ and implied in the previous section to explain the evolution of the spectra. The SIQR of ^23^Na increases with the basification of the medium due to two main factors. On one hand, higher pH values lead to the ionization of a greater proportion of surface hydroxyls (i.e., more adsorption sites ≡M–O^*–*^ for Na^+^ ions), and the instantaneous fraction of adsorbed ^23^Na becomes larger, resulting in greater overall SIQR effects. Conversely, acidification of the medium creates more positively charged adsorption sites (M–OH_2_^+^) for Br^–^ ions. On the other hand, the magnitude of the EFG is likely to increase with surface charge density.^36^ Since surface ionization intensifies with changes in pH, there is strong evidence to expect surface-induced EFG to increase for adsorbed counterions, causing the relaxation rate *R*_1,surf_ while adsorbed to also become larger (see Equation 6). Interestingly, the SIQR effect of ^23^Na^+^ and ^81^Br^–^ in positive and negative surface charge pH regions is almost undetectable (i.e., their relaxation rates are close to those in the pure electrolyte), indicating that they are repelled by the surfaces and that very few negative or positive charges, respectively, are present on the surface in these pH ranges.

**Figure 7:**
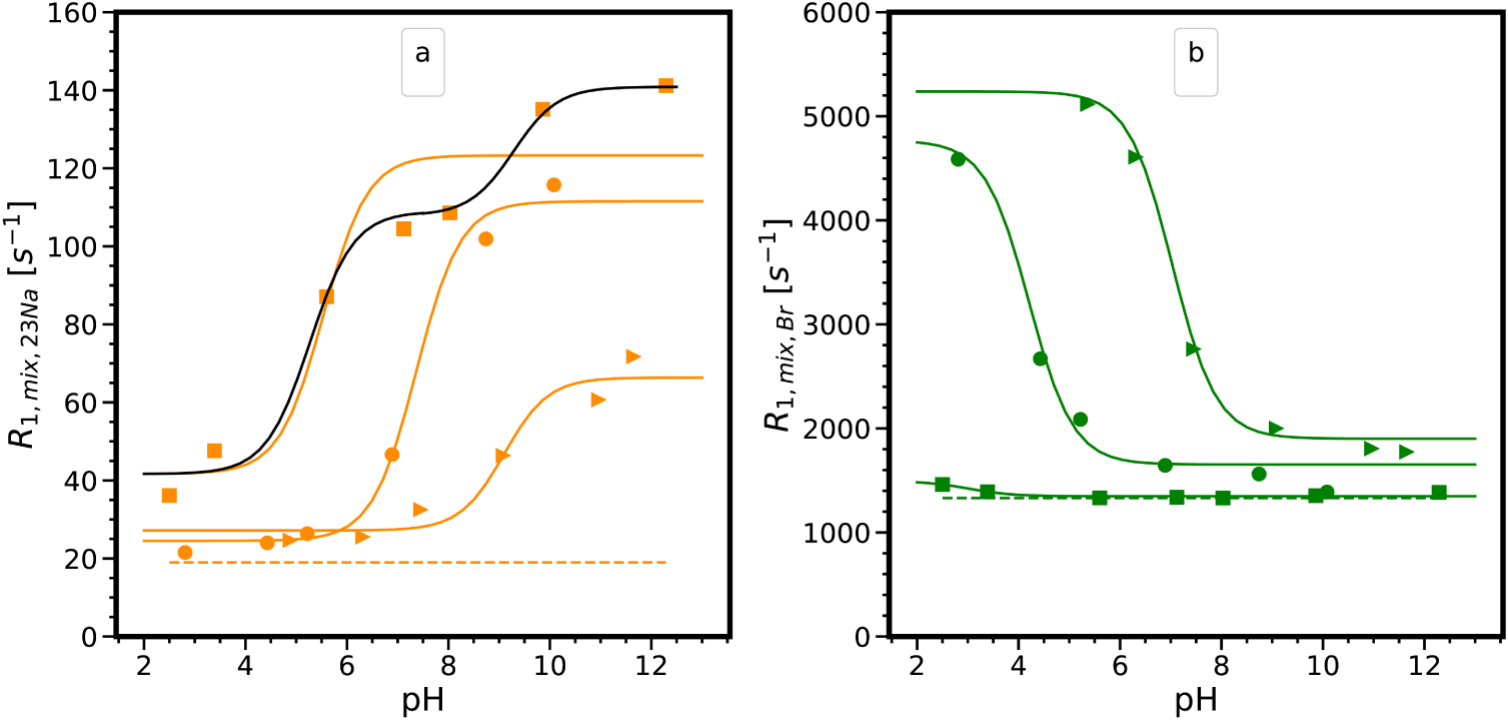
Evolution of ^23^Na (a) and ^81^Br (b) longitudinal relaxation rates (*R*_1_ = 1/*T*_1_) as a function of pH in mixtures involving TiO_2__325 (squares), TiO_2__P25 (circles), and AKP-30 (triangles). The full colored lines are best fits obtained with the modified Henderson–Hasselbalch equation with one p*K*_a_ value, and the black line corresponds to the fit with two p*K*_a_ values for TiO_2__325. The dotted lines represent the relaxation rates in the bulk electrolytes.

Regarding TiO_2__325, the trend of the ^23^Na SIQR curve is similar to those of TiO_2__P25 and AKP-30, indicating an increase in the density of negative charges on the surface, while no ^81^Br SIQR effect is detected over the investigated pH range, indicating that Br^–^ ions never adsorb on the TiO_2__325 surface and no positive charges are created even at our lowest pH values. This evolution is consistent with the acidic nature of TiO_2__325 and is supported by our zeta potential measurements. These acidic surface groups ≡M–OH cannot be converted into ≡M–OH_2_^+^; no Br^–^ counterions will get close enough to the surface to experience SIQR. This is expected if the surface presents a significant proportion of phosphate groups, which will hardly become positively charged over this pH range.

Based on our interpretation of SIQR effects, TiO_2__325 is the oxide with the highest affinity for Na^+^ ions, followed by TiO_2__P25 and AKP-30. On the other hand, TiO_2__P25’s affinity for Br^–^ ions is the strongest in the region of positive surface charges, followed by AKP-30 and TiO_2__325. This hierarchy is again consistent with the order displayed in the zeta potential curves (Figure 3) in the two regions. As expected, the oxide with the highest net surface charge is the oxide with the strongest SIQR.

The acid/base properties of surface groups (p*K*_a_) and the isoelectric points (IEPs) are often used to characterize the physicochemical properties of oxide surfaces. Assuming a single average value, the p*K*_a_ and IEP were determined from the modified Henderson–Hasselbalch equation:

7 $$pH=pKa+\log\left(\frac{\Delta R_{1}-a}{b-\Delta R_{1}}\right)$$

In this equation, the SIQR effect is noted Δ*R*_1_ = *R*_1,mix_ – *R*_1,bulk_, *a* represents the value of Δ*R*_1_ at maximum surface protonation, and *b* represents the value of Δ*R*_1_ when the surface is completely deprotonated. In its original form, the Henderson–Hasselbalch equation was written to relate the pH of a weak acid to the acidity constant (*K*_a_) and the ratio of the activities of the acid and its conjugate base (i.e., $\frac{\left[base\right]}{\left[acid\right]}$ ) in the medium.^76^ It was first adapted to express the dependence of NMR chemical shifts on pH,^77,78^ before being extended to account for changes in the relaxation rates. Equation 7 is inspired by the one written by Pollard et al.^79^ to describe the pH dependence of surface relaxivity. In practice, Equation 7 was used to simulate our SIQR curves as a function of pH (Figure 7). Overall, the modified Henderson–Hasselbalch equation provides a good description of the SIQR effect for both ^23^Na and ^81^Br in TiO_2__P25 (*R* ^2^ = 0.988 and 0.977, respectively) and AKP-30 (*R^2^* = 0.93 and 0.975, respectively) with a single p*K*_a_ value for each curve. For TiO_2__325, both ^23^Na and ^81^Br fits with single p*K*_a_ values are of poorer quality, with *R* ^2^ = 0.837 and *R* ^2^ = 0.767, respectively. Concerning ^81^Br relaxation, as no significant SIQR effect is detected, we assumed that the surface was hardly ever protonated. Referring to the FT-IR spectra (Supporting Information, Section C) and the detection of ^1^H and ^31^P NMR signals for TiO_2__325, its ^23^Na SIQR curve was simulated assuming that the surface groups were negatively charged weak diacids (one p*K*_a_ for each acidity), a behavior consistent with a mix of carbonate and/or phosphate moieties. This led to a much better fit with p*K*a_1_ = 5.7 and p*K*a_2_ = 9.3 (Figure 7), supporting the key role of multiple groups with distinct p*K*_a_ in TiO_2__325’s behavior.

Table 2 presents the optimized parameters from the modified Henderson–Hasselbalch equation. Regarding TiO_2__P25 and AKP-30, IEP values of 5.8 and 8.1, respectively, have been estimated (Equation 3), compared to 7.1 and 8.8 from zeta potential experiments, respectively. These values are of the same order of magnitude, although they were not obtained under the same conditions. As a reminder, the SIQR experiments were carried out on compact slurry-like samples with an ionic strength of 1 M, while the zeta potential measurements mentioned are those obtained on colloidal samples at 0.1 M ionic strength. Moreover, the solid contents were 41, 72, and 86 wt % for TiO_2__P25, TiO_2__325, and AKP-30, respectively, compared to 3 wt % for the zeta potential measurements. The analysis of the SIQR effect offers an alternative to zeta potential measurements for large solid contents and high ionic strengths, where the latter is usually less reliable. On the other hand, SIQR experiments require high ionic strengths to avoid limitations related to sensitivity and problems with magnetization inversion or saturation and perform better in media with relatively large solid content to ensure the SIQR affects enough ions. The small deviation between the IEP_NMR_ and the IEP_zeta_ can be attributed to either the influence of solid content or the ionic strength, as shown in our work (IEP of TiO_2__P25 going from 7.1 to 4.5 for samples with 0.1 and 1 M NaBr solutions) and previous studies stating that large solid content or higher ionic strengths can modify p*K*_a_ values^76^ and therefore IEP.^4^

**Table 2:** Optimization Parameters (*a*, *b*, and p*K*_a_) Used for pH-Dependent SIQR Curves Fitted with the Modified Henderson–Hasselbalch Equation^a^

Nuclei	Parameters	TiO_2__P25	TiO_2__325	pH < 8.03	pH > 8.03	AKP-30
^81^Br	*a*_1_	3436 ± 204	164 ± 58			3906 ± 81
	*b*_1_	322 ± 98	16 ± 10.2			570 ± 47
	p*K*a_1_	4.2 ± 0.1	3.0 ± 0.52			7.0 ± 0.1
^23^Na	*a*_2_	5.6 ± 2.7	22.7 ± 11	22.3	89.6	8.2 ± 3.2
	*b*_2_	92 ± 4	104 ± 14	89.6	122.6	47 ± 4
	p*K*a_2_	7.4 ± 0.2	5.5 ± 0.4	5.70 ± 0.14	9.28 ± 0.18	9.1 ± 0.3
**IEP_NMR_**		5.8 ± 0.3	-			8.1 ± 0.4
**IEP**_zeta_		7.05	-			8.8

a The IEPs calculated from these p*K*a_i_ values (IEPNMR) for TiO_2__P25 and AKP-30 are compared to those from the zeta potential obtained in 0.1M colloidal suspensions (IEP_zeta_).

## Conclusion

In this work, we developed a straightforward relaxometry-based approach to probe the surface electric properties of oxides in aqueous environments, particularly under conditions where conventional zeta potential measurements become unreliable—namely, at high ionic strengths (≥0.1 M) and in dense, noncolloidal suspensions (up to 86 wt %). We demonstrated that under these conditions, zeta potential curves are significantly distorted and require background correction. For example, in 1 M suspensions of TiO_2__P25, zeta potential curves were drastically altered, with no discernible isoelectric points (IEPs). A single background correction for this system shifted the IEP from 7.05 (observed at 0.1 M and 3 wt %) to 4.53. In contrast, background corrections failed for TiO_2__325 and AKP-30 under the same conditions.

Remarkably, the relaxometry-based protocol remained effective in these challenging regimes. By correlating pH-dependent surface-induced quadrupolar relaxation effects (SIQR) with zeta potentials, we gained new insights into the surface charge distribution of the oxides. Specifically, we exploited the selective sensitivity of ^81^Br and ^23^Na nuclei to detect positive and negative surface charges, respectively, providing information complementary to that obtained from traditional zeta potential measurements. This approach enabled a qualitative description of the net surface charge for TiO_2__P25, TiO_2__325, and AKP-30 as a function of pH (provided *R*_1,surf_ changes with pH), specifically in compact and highly concentrated suspensions, which are impractical conditions for zeta measurements and are commonly encountered in many areas of chemistry.

Furthermore, modeling the relaxation behavior using a modified Henderson–Hasselbalch equation allowed us to estimate the IEP for TiO_2__P25 and AKP-30 in dense sludges (41 and 86 wt %) at 1 M ionic strength, yielding values of 5.8 and 8.1, respectively. These values are consistent with the IEP obtained from dilute (0.1 M) colloidal suspensions, reinforcing the validity of the SIQR-based method. In the case of TiO_2__325, its exclusively acidic behavior was attributed to different surface groups, including phosphate and carbonyl moieties.

Despite its advantages, the SIQR approach faces certain limitations. Fast relaxation during RF pulsing can impact measurement accuracy and must be considered during protocol optimization. Additionally, manual sample preparation introduces variability, unlike fully automated techniques such as acoustophoresis. Therefore, future developments should focus on automating the method to enhance its reproducibility and minimize sources of error.

The ability to characterize surface properties in highly concentrated electrolytes and dense solid suspensions holds relevance for fields such as heterogeneous catalysis, electrochemistry, electrochemical energy storage, colloid and interface science, environmental chemistry, and materials synthesis, where interfacial phenomena critically influence reactivity, stability, and functional performance.

## Supplementary Material



## Notes

Scanning electronic microscopy images (Section A); thermogravimetric analysis (Section B); solid content determination procedure (Section C); Fourier-transformed infrared spectroscopy spectra (Section D); magnetization evolution curves (Section E) for TiO_2__P25, TiO_2__325, and AKP-30 (PDF)
