# Supplementary material for: Cotracking of Na+ and Br– Adsorption through Surface-Induced Quadrupolar Relaxation (SIQR): An Alternative to Zeta Potential Measurements for High Ionic Strengths (>0.1 M) and Nondispersed Liquid–Solid Mixtures
Source: J Phys Chem A. 2025 Sep 15;129(40):9412–22. doi: 10.1021/acs.jpca.5c02542 (PMC12516720; doi:10.1021/acs.jpca.5c02542)
Supplement: Supplementary file 1 [file jp5c02542_si_001.pdf]

Co-tracking of Na^+ and Br^- adsorption through Surface Induced Quadrupolar Relaxation (SIQR): an alternative to zeta potential measurements for high ionic strengths (>0.1 M) and non-dispersed liquid-solid mixtures

Zlanseu Ruth Tan, [a] *Cécile Pagnoux,* [b] *Vincent Sarou-Kanian* [a, *], *Sandra Ory* [a] *and Michaël Deschamps* [a, *]

[a] CEMHTI, CNRS UPR 3079, Université d'Orléans, F-45071 Orléans, France

[b] IRCER, Centre Européen de la Céramique, Université de Limoges, 12 rue Atlantis, 87068 Limoges, France

KEYWORDS: adsorption, zeta potential, surface, NMR, quadrupolar, relaxation

SUPPLEMENTARY INFORMATION

A. Scanning Electronic Microscopy (SEM) analysis

On the SEM images below (Figure S1) grains appear as agglomerates of non-uniform sizes, whatever the oxide. The size ranges estimated from the images are 25-51 nm, 70-238 nm and 87-814 nm for TiO_2_P25, TiO_2_325 and AKP-30, respectively. The best way would have been to perform a laser granulometry analysis (which is beyond the scope of this work) in order to confirm the literature on TiO_2_P25 and AKP-30 and to provide primary grain size for TiO_2_325. The present estimation remains a qualitative overview of grain sizes distribution.

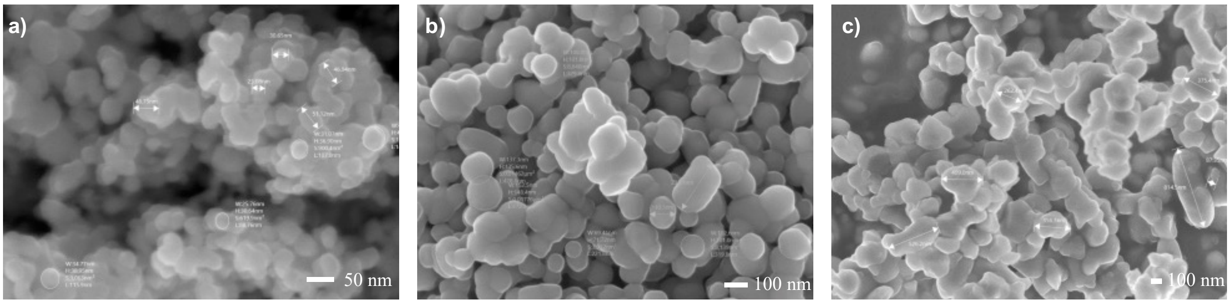

Figure S1: Scanning Electron Microscopy images of a) TiO_2_P25, b) TiO_2_325 and c) α-Al_2O_3 (AKP-30).

B. Thermogravimetric analysis (TGA)

Prior to mixtures preparation, oxides were submitted to thermogravimetric analysis (TGA) to quantify the surface -OH density (σ_{OH}) in numbers of OH goups/nm^2. Experiments were performed with a SETARAM thermobalance (ATG/ATD Setsys Evo 2400), over a temperature ranging from 25°C to 500°C. For each oxide, a known amount was heated under an argon atmosphere (20 mL/min) up to 500°C, at 10 °C/min. The sample was held at this temperature for 10 minutes before returning to room temperature. The weight change was

recorded as a function of temperature and converted into relative weight loss (Wt%). The weight losses were identified accurately on all thermograms using first derivative curves. For samples with weight losses in the temperature range attributed to the -OH removal, σ_{OH} was calculated through the following equation:

$$\sigma_{OH} = \frac{Wt\% \times N_A}{100 \times M_{OH} \times SSA} \quad (1)$$

Where N_A is the Avogadro constant ($6.022 \times 10^{23} mol^{-1}$), M_{OH} is the molar mass of an -OH group (17 g/mol) and SSA is the specific surface area (nm^2/g). This formula was derived from the work of Mueller and co-workers.[1]

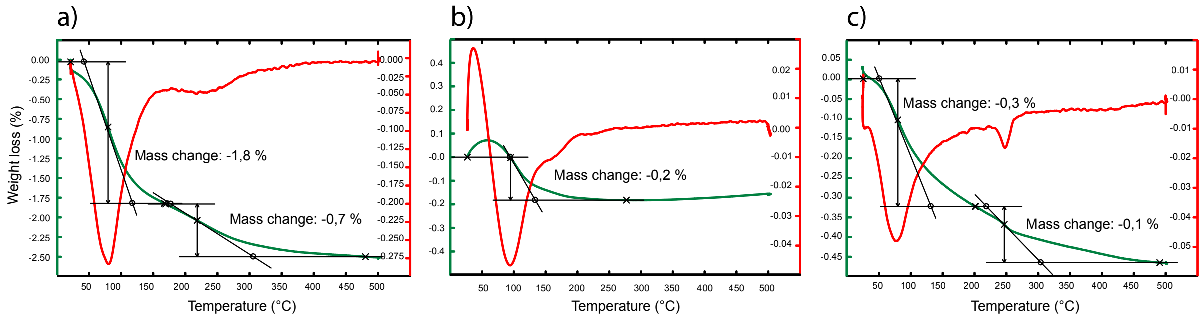

Figure S2: Thermogravimetric analysis (TGA) of a) TiO_2_P25, b) TiO_2_325 and c) α-Al_2O_3 (AKP-30). The black lines are the measured weight loss profiles, and the red lines are the resulting first derivative curves. The measurements were performed under an argon flow of 20 mL/min from room temperature to 500°C, at a heating rate of 10 °C/min.

Figure S2 represents the TGA thermograms obtained for TiO_2_P25, TiO_2_325 and AKP-30. The green lines are the weight loss profiles, and the red lines are the first derivative curves that emphasize the temperature ranges of mass losses. In the case of TiO_2_P25 and AKP-30, two weight losses are recorded whereas only one is observed for TiO_2_325. The weight losses of 1.8% (25-170 °C), 0.3% (25-200 °C) and 0.2% (25-250 °C) for respectively TiO_2_P25, AKP-30 and TiO_2_325 have been associated with the desorption of weakly bound water

molecules (i.e. dehydration). Regarding the second losses for TiO_2_P25 (0.7% at 170-500 °C) and AKP-30 (0.1% at 200-500 °C), they were assigned to the release of surface OH groups (dehydroxylation). These attributions were in good agreement with those reported in literature for TiO_2 (P25 and others).[1–3] The surface -OH densities has been calculated from the weight losses using equation 6 of the main paper. The resulting densities were respectively 4.9 OH/nm^2 and 5.3 OH/nm^2 for TiO_2_P25 and AKP-30, respectively. The value for TiO_2_P25 was close to that determined by Mueller et al. (4.8 OH/nm^2)[1] and slightly lower than that of Wu et al. (5.3 OH/nm^2).[2] Concerning AKP-30, we found only one reference to the -OH density in a work by Pagnoux et al.[4] with a reported value of 10 OH/nm^2. The later was deduced from the pH-dependent zeta potential curve fitting, which could justify the difference with our experimental 5.3 OH/nm^2 value. The -OH density calculation was made solely for TiO_2_P25 and AKP-30, since TiO_2_325 showed no evidence of dehydroxylation over the relevant temperature range. This suggests an almost complete absence of surface -OH groups for this material, assuming the maximum operating temperature of 500°C is sufficient for its dehydroxylation.

C. Sample solid contents

The solid content of our slurries was difficult to determine accurately, as the samples were filtrated under vacuum, without any possibility to control the amount of solution removed. All mixtures were initially loaded with around 3 wt% oxides. Table 1 shows the values of pH_0, pH_mix and sediment masses obtained after equilibration and filtration of each mixture, depending on the oxide and pH. As expected, the sediment masses were almost identical in all the series. The slight variations observed for TiO_2_P25 and AKP-30 were considered negligible

in our interpretations. Therefore, the sediment solid content was derived by considering the following ratio:

$$W(\%) = 100\frac{m_{oxide}}{m_{sediment}} \tag{2}$$

In our approach, we ignored the possible material loss due for example to the residues remaining in containers after filtration. We assumed that all the oxide previously added in the mixtures were recovered after the filtration and that no significant evaporation occured. The oxide contents were around 41, 72 and 86 wt% in mixtures involving TiO_2_P25, TiO_2_325 and AKP-30, respectively, after filtration. These values are average of the solid contents obtained over the entire pH range for each oxide.

Table 1: Initial pH (pH_0) of 1 mol/L aqueous NaBr before adding to oxides, pH (pH_mix) of mixtures after equilibration and mass of sediment ($m_{sediment}$) obtain for each pH, after filtration.

Material	pH_0	pH_mix	$m_{sediment}$ (g)
TiO_2_P25	2.33	2.8	1.1
	2.9	4.4	1.2
	4.0	5.2	1.3
	11.1	6.9	1.3
	11.6	8.7	1.2
	12.1	10.1	1.3
TiO_2_325	2.3	2.5	1.7
	2.9	3.4	1.7
	5.9	5.6	1.7
	10.3	7.1	1.7
	11.0	8.0	1.7
	11.3	9.9	1.7
	12.4	12.3	1.7

AKP-30	3.3	5.4	1.5
	3.5	6.3	1.5
	9.0	7.4	1.4
	11.3	9.1	1.6
	11.5	10.9	1.5
	11.7	11.6	1.4

D. Fourier Transformed Infrared Spectroscopy (FTIR)

In addition to TGA, Infrared (IR) spectroscopy experiments were performed to confirm the presence of -OH groups at oxide particles surface and to identify potential surface impurities. Spectra were recorded in transmittance mode, with an infrared Fourier-transform spectrometer (FT-IR) Vertex V70 from Bruker. These analyses were performed on oxide/KBr pellets in a ratio of 1:1000, for TiO_2_325 and 1:100 for both TiO_2_P25 and AKP-30, over a wavenumber range from 350 to 4000 cm^{-1}.

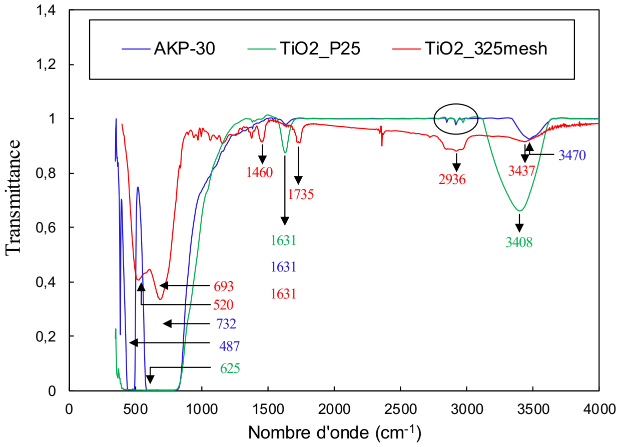

Figure S3: Infrared spectra of TiO_2_P25 (green), TiO_2_325 (red) and α-Al_2O_3 (blue).

The most obvious observation remains the complexity of TiO_2_325 spectrum compared to those of TiO_2_P25 and AKP-30, due to its greater number of bands (Figure S3).

On the TiO_2 spectra, the characteristic Ti-O and O-Ti-O stretching and bending vibrations appear at 423-625 cm^{-1} for TiO_2_P25 and at 520 cm^{-1} and 693 cm^{-1} for TiO_2_325. The bands at 1631 cm^{-1} (for both), 3408 cm^{-1} (TiO_2_P25) and 3437 cm^{-1} (TiO_2_325) are ascribed respectively to H-OH bending and -OH stretching vibrations.

For AKP-30, the classical absorption bands appear at 487 cm^{-1} and 732 cm^{-1} for AlO_6 bending and stretching, at 1631 cm^{-1} and 3470 cm^{-1} for H-OH bending and -OH stretching modes, respectively.

In addition to these characteristic adsorption bands, TiO_2_325 presents some organic adsorption bands at 1460 cm^{-1}, 1379 cm^{-1} and 2936 cm^{-1}, attributed to C-H bending, symmetric deformation and stretching respectively.

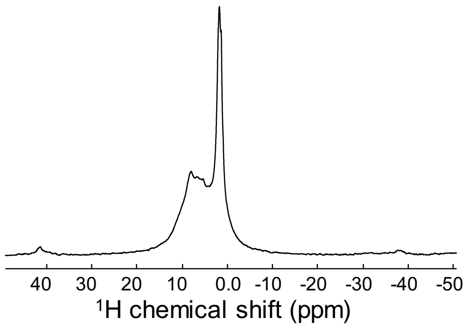

Figure S4: ^{1}H Hahn echo NMR spectrum of TiO_2_325. The spectrum was recorded on a Bruker 200 MHz WB spectrometer equipped with a 4 mm ^{1}H/X double resonance probe and with Magic Angle Spinning at 8 kHz, a 1 second recycling delay and 256 transients.

This assignment has been confirmed by ^{1}H NMR (Figure S4), which showed a significant peak around 1.7 ppm (which corresponds to the range of chemical shift for saturated alkane R-H). The additional band at 1735 cm^{-1} for this material was assigned to C=O stretching.

This reflects the presence of organic moieties on the TiO_2_325 surface, as seen in anatase TiO_2 polymorph.[5,6] Such alkane and carboxylate groups generally come from the synthesis organic precursors.[5] The TiO_2_325 spectrum also shows strong evidence of phosphate groups on the surface, with low-intensity vibration bands at 1259 cm^{-1} (P=O stretching), 1168 cm^{-1} and 970 cm^{-1} (P-O stretching) and 840 cm^{-1} (Ti-O-P stretching).[7–10] This is consistent with our ^{31}P NMR experiment which confirmed the presence of phosphate groups in the sample (Figure S5).

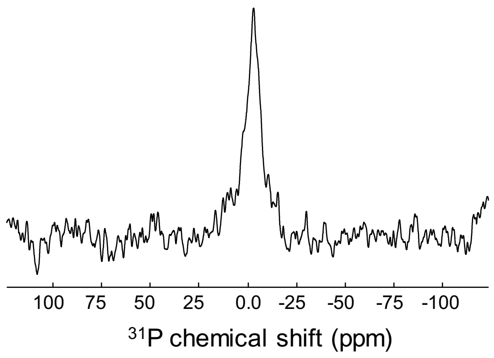

Figure S5: ^{31}P single pulse spectrum of TiO_2_325. The spectrum was recorded on a Bruker 200 MHz WB spectrometer equipped with a 4 mm $^{1}H/X$ double resonance probe tuned to ^{31}P and with Magic Angle Spinning at 8 kHz, a 1 second recycling delay and 4096 transients. The 90° pulse length was 3.76 μs for a power of 80 W.

E. Evolution of spectra during the inversion-recovery experiments

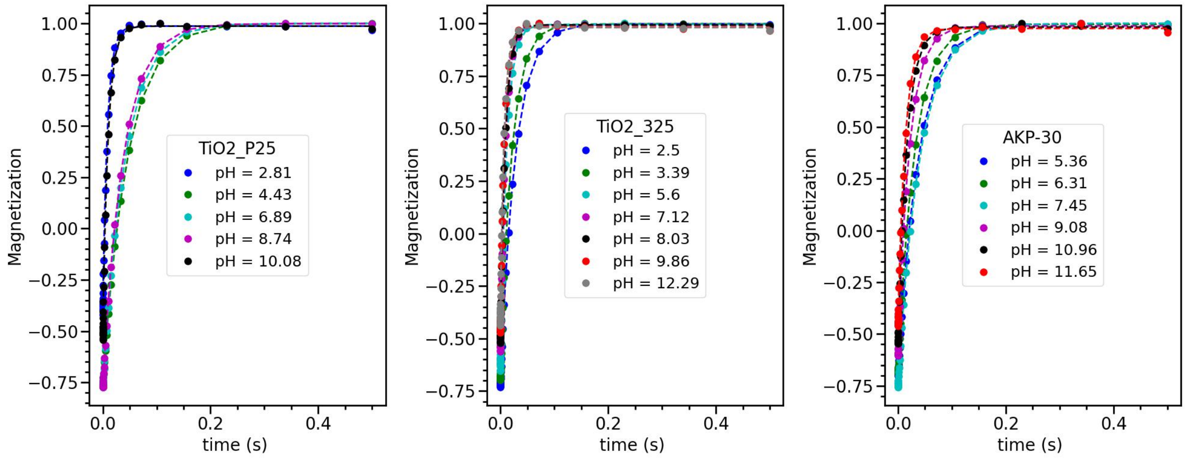

Figure S6: ^{23}Na magnetization curves as a function of pH in solid/liquid mixtures involving TiO_2_P25 (left), TiO_2_325 (center) and AKP-30 (right).

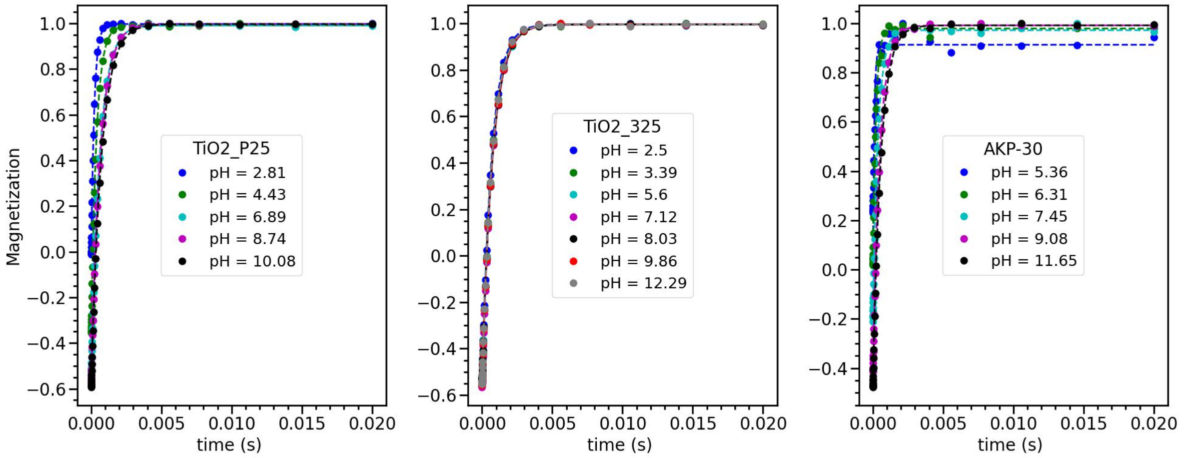

Figure S7: ^{81}Br magnetization curves as a function of pH in solid/liquid mixtures involving TiO_2_P25 (left), TiO_2_325 (center) and AKP-30 (right).

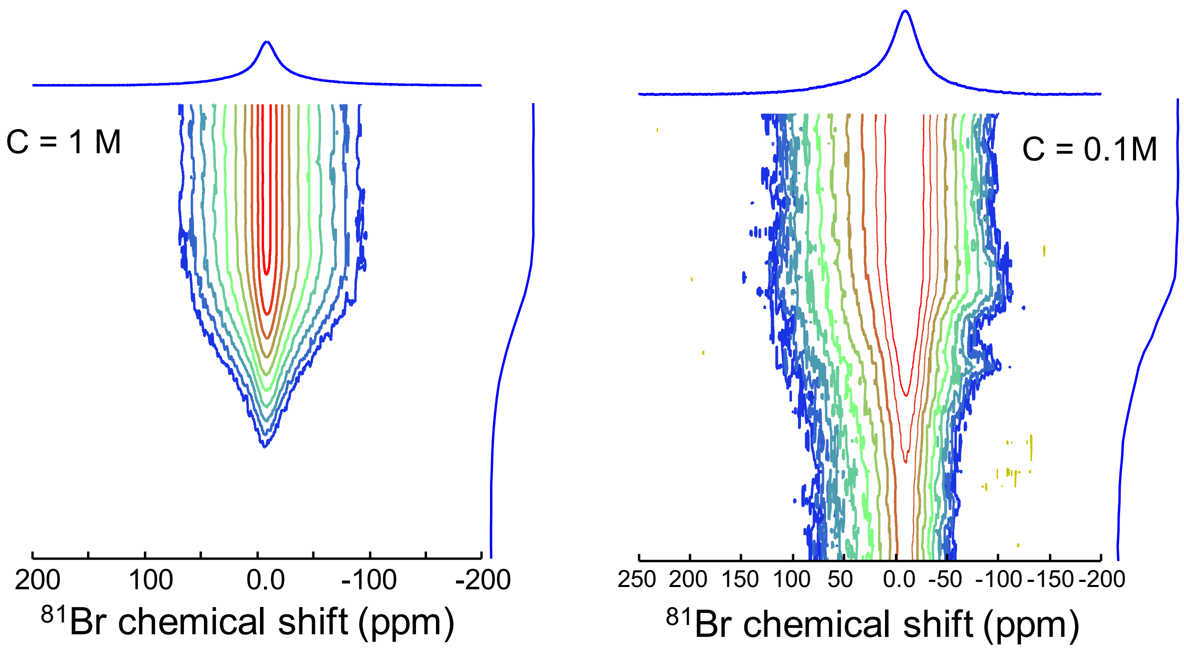

Figure S8: ^{81}Br inversion-recovery contour plots for mixtures involving TiO2_P25 at 1 mol/L (left) and 0.1 M (right). A small proportion of the signal is inversed in the 1 *M* sample while no inversion is observed at 0.1 *M*. The *pH* values were recorded at 9.6 and 2.9 for the 1 *M* and 0.1 *M* samples respectively.

F. Linewidths as a function of pH

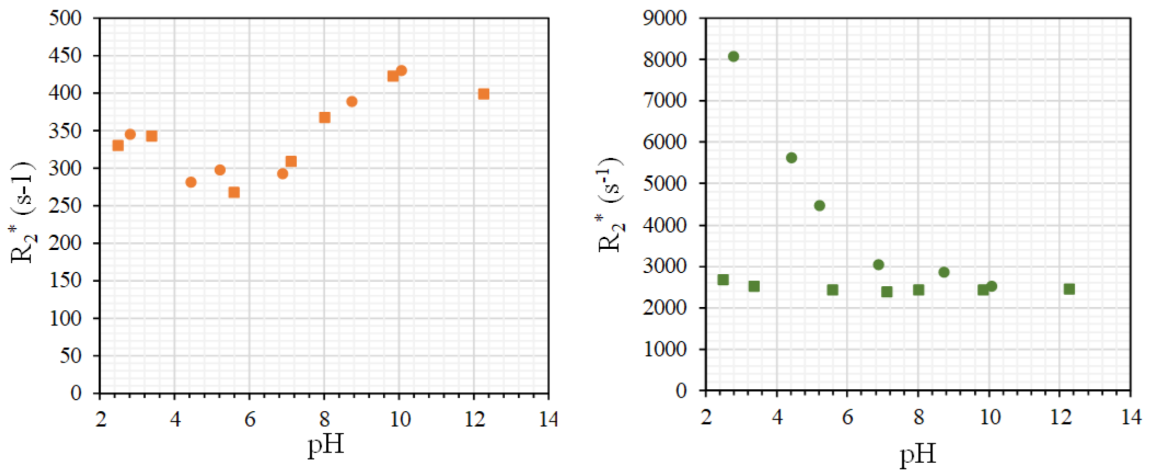

Figure S9. Full width at half maximum for ^{23}Na (orange) and ^{81}Br (green) for TiO_2_P25 (circles) and TiO_2_325 (squares), measured from the fully relaxed 1D spectra using the peak picking function of the Bruker Topspin software.

For both materials, the ^{23}Na R_2^* curves overlap with a decrease of R_2^* values below pH 6 and an increase above.

For this nucleus, the contribution of the magnetic field inhomogeneity to the linewidth is possibly quite large, which could justify the overlapping of the curves and the loss of materials specificities. Therefore, interpreting the ^{23}Na R_2^* curves may be difficult. Regarding ^{81}Br, the R_2^* curve trends are like those presented in the main text for R_1. The curve for TiO_2_325 presents little variation over the entire pH range while the relaxation of TiO_2_P25 is enhanced in the acidic pH region. In this case, the linewidths are probably dominated by relaxation phenomena, and inhomogeneity effects are negligible, so that R_2^* can be used in place of R_1 measurements in these specific cases.

As expected, whatever the nucleus and the material, the R_2^* values are higher than the R_1 values presented in the manuscript. Moreover, at solid/liquid interfaces, ions are probably out of the extreme narrowing regime, where transverse relaxation is expected to be biexponential, with relaxation induced lineshapes that are described by two Lorentzian lines in the best cases, and therefore the FWHM is likely not sufficient to describe the linewidth precisely.

Bibliography

(1) Mueller, R.; Kammler, H. K.; Wegner, K.; Pratsinis, S. E. OH Surface Density of SiO_2 and TiO_2 by Thermogravimetric Analysis. *Langmuir* **2003**, *19* (1), 160–165. https://doi.org/10.1021/la025785w.

(2) Wu, C.-Y.; Tu, K.-J.; Deng, J.-P.; Lo, Y.-S.; Wu, C.-H. Markedly Enhanced Surface Hydroxyl Groups of TiO_2 Nanoparticles with Superior Water-Dispersibility for Photocatalysis. *Materials* **2017**, *10* (5), 566. https://doi.org/10.3390/ma10050566.

(3) Di Paola, A.; Bellardita, M.; Palmisano, L.; Barbieriková, Z.; Brezová, V. Influence of Crystallinity and OH Surface Density on the Photocatalytic Activity of TiO_2 Powders. *Journal of Photochemistry and Photobiology A: Chemistry* **2014**, *273*, 59–67. https://doi.org/10.1016/j.jphotochem.2013.09.008.

(4) Pagnoux, C.; Serantoni, M.; Laucournet, R.; Chartier, T.; Baumard, J.-F. Influence of the Temperature on the Stability of Aqueous Alumina Suspensions. *Journal of the European Ceramic Society* **1999**, *19* (11), 1935–1948. https://doi.org/10.1016/S0955-2219(99)00007-2.

(5) Praveen, P.; Viruthagiri, G.; Mugundan, S.; Shanmugam, N. Structural, Optical and Morphological Analyses of Pristine Titanium Di-Oxide Nanoparticles – Synthesized via Sol–Gel Route. *Spectrochimica Acta Part A: Molecular and Biomolecular Spectroscopy* **2014**, *117*, 622–629. https://doi.org/10.1016/j.saa.2013.09.037.

(6) Filippo, E.; Carlucci, C.; Capodilupo, A. L.; Perulli, P.; Conciauro, F.; Corrente, G. A.; Gigli, G.; Ciccarella, G. Enhanced Photocatalytic Activity of Pure Anatase TiO_2 and Pt-TiO_2 Nanoparticles Synthesized by Green Microwave Assisted Route. *Mat. Res.* **2015**, *18* (3), 473–481. https://doi.org/10.1590/1516-1439.301914.

(7) Colthup, N. B.; Daly, L. H.; Wiberley, S. E. *Introduction to Infrared and Raman Spectroscopy*, 2d ed.; Academic Press: New York, 1975.

(8) Silverstein - Spectrometric Identification of Organic Compounds 7th Eds.

(9) Pivkina, A. N.; Muravyev, N. V.; Monogarov, K. A.; Fomenkov, I. V.; Schoonman, J. Catalysis of HMX Decomposition and Combustion. In *Energetic Nanomaterials*; Elsevier, 2016; pp 193–230. https://doi.org/10.1016/B978-0-12-802710-3.00009-X.

(10) Zhao, D.; Chen, C.; Wang, Y.; Ji, H.; Ma, W.; Zang, L.; Zhao, J. Surface Modification of TiO_2 by Phosphate: Effect on Photocatalytic Activity and Mechanism Implication. *J. Phys. Chem. C* **2008**, *112* (15), 5993–6001. https://doi.org/10.1021/jp712049c.
